# Supplementary material for: Anti-Inflammatory Activity of Gomphrenin-Rich Fraction from Basella alba L. f. rubra Fruits
Source: Nutrients. 2024 Dec 20;16(24):4393. doi: 10.3390/nu16244393 (PMC11678042; doi:10.3390/nu16244393)
Supplement: Supplementary file 1 [file nutrients-16-04393-s001.zip › Nutrients -3361835-Supplementary_Files/Table S1.docx]

| **Cohen’s d effect size referring to specified substance/agent** | | | | | | | | | | |
| --- | --- | --- | --- | --- | --- | --- | --- | --- | --- | --- |
|  | **MDA** | **COX** | **DCF-DA** | **GRIESS** | **IL-1β** | | **IL-6** | | **NfκB** | |
| **Ref CMPD** | **SIN-1** | **LPS-1h** | **H_2_O_2_** | **SIN-1** | **M** | **LPS-1h** | **M** | **LPS-1h** | **M** | **LPS-1h** |
| **LPS 1h** | 8.66 | - | 3.62 | 2.33 | 1.47 | - | 23.01 | - | 3.02 | **-** |
| **LPS 24h** | 5.39 | 0.69 | 1.34 | 4.72 | 3.87 | 1.37 | 42.11 | 1.24 | 4.79 | 2.67 |
| **A 0.1** | 2.24 | 8.99 | 7.16 | 17.87 | 5.09 | 1.59 | 1.85 | 22.30 | 5.16 | 2.26 |
| **A 1** | 11.26 | 16.74 | 7.71 | 38.98 | 4.39 | 1.58 | 2.45 | 16.79 | 6.04 | 3.31 |
| **A 10.0** | 14.76 | 18.76 | 13.37 | 33.89 | 4.75 | 1.58 | 10.48 | 23.79 | 5.21 | 2.27 |
| **B 0.1** | 2.47 | 21.30 | 5.57 | 30.26 | 5.12 | 1.59 | 7.56 | 23.57 | 5.23 | 2.97 |
| **B 1** | 10.31 | 19.22 | 12.76 | 7.26 | 5.09 | 1.59 | 0.50 | 23.08 | 4.35 | 1.66 |
| **B 10.0** | 13.21 | 14.74 | 18.03 | 13.35 | 4.96 | 1.59 | 0.30 | 23.03 | 4.52 | 1.44 |
| **Mel 0.1** | 10.90 | 16.55 | 5.13 | 33.75 | 3.50 | 1.55 | 3.53 | 23.37 | 10.95 | 9.67 |
| **Mel 1** | 13.33 | 13.48 | 8.34 | 37.57 | 5.35 | 1.60 | 11.58 | 23.79 | 5.93 | 3.76 |
| **Mel 10.0** | 14.95 | 20.63 | 13.38 | 24.10 | 4.53 | 1.58 | 2.24 | 23.20 | 5.61 | 4.12 |
| **Ind 0.1** | 5.50 | 18.22 | 5.99 | 23.05 | 3.01 | 1.54 | 0.66 | 22.89 | 15.13 | 14.88 |
| **Ind 1** | 14.80 | 18.43 | 7.34 | 47.42 | 4.49 | 1.58 | 1.56 | 22.01 | 6.09 | 4.41 |
| **Ind 10.0** | 14.56 | 19.73 | 14.06 | 35.59 | 4.03 | 1.57 | 0.85 | 22.88 | 4.51 | 1.27 |
| **Asc 0.1** | 8.11 | - | 9.18 | 36.83 | 4.39 | 1.58 | 2.99 | 22.58 | 0.73 | 3.70 |
| **Asc 1** | 10.66 | - | 10.73 | 37.92 | 4.51 | 1.58 | 4.68 | 23.43 | 2.51 | 0.96 |
| **Asc 10.0** | 1.91 | - | 9.32 | 31.31 | 4.43 | 1.58 | 1.10 | 22.98 | 2.68 | 8.07 |
| **Asp 0.1** | - | 12.07 | - | - | - | - | - | - | - | - |
| **Asp 1** | - | 21.14 | - | - | - | - | - | - | - | - |
| **Asp 10.0** | - | 20.67 | - | - | - | - | - | - | - | - |

Table S1. The confidence intervals (CIs) of the effect sizes showing the strength of the relationship between the tested compounds and control/reference compounds.

M- medium

A – betalain enriched fraction

B – crude extract

Mel – meloxicam

Ind – indomethacin

Asc - ascorbic acid

Asp – aspirin

LPS-1h – lipopolysaccharide 1-hour exposition

LPS-24h – lipopolysaccharide 24-hour exposition

SIN-1 - peroxynitrite generator

H_2_O_2_ – hydrogen peroxide

The concentration range of the tested substances was given in μg/ml.
